# Defining Terms Used for Animals Working in Support Roles for People with Support Needs

**DOI:** 10.3390/ani12151975

**Published:** 2022-08-04

**Authors:** Tiffani J. Howell, Leanne Nieforth, Clare Thomas-Pino, Lauren Samet, Sunday Agbonika, Francisca Cuevas-Pavincich, Nina Ekholm Fry, Kristine Hill, Brinda Jegatheesan, Miki Kakinuma, Maureen MacNamara, Sanna Mattila-Rautiainen, Andy Perry, Christine Y. Tardif-Williams, Elizabeth Ann Walsh, Melissa Winkle, Mariko Yamamoto, Rachel Yerbury, Vijay Rawat, Kathy Alm, Ashley Avci, Tanya Bailey, Hannah Baker, Pree Benton, Catherine Binney, Sara Boyle, Hagit Brandes, Alexa M. Carr, Wendy Coombe, Kendra Coulter, Audrey Darby, Lowri Davies, Esther Delisle, Marie-Jose Enders-Slegers, Angela Fournier, Marie Fox, Nancy Gee, Taryn M. Graham, Anne Hamilton-Bruce, Tia G. B. Hansen, Lynette Hart, Morag Heirs, Jade Hooper, Rachel Howe, Elizabeth Johnson, Melanie Jones, Christos Karagiannis, Emily Kieson, Sun-A Kim, Christine Kivlen, Beth Lanning, Helen Lewis, Deborah Linder, Dac Loc Mai, Chiara Mariti, Rebecca Mead, Gilly Mendes Ferreira, Debbie Ngai, Samantha O’Keeffe, Grainne O’Connor, Christine Olsen, Elizabeth Ormerod, Emma R. Power, Peggy A. Pritchard, Kerri Rodriguez, Deborah Rook, Matthew B. Ruby, Leah Schofield, Tania Signal, Jill Steel, Wendy Stone, Melissa Symonds, Diane van Rooy, Tiamat Warda, Monica Wilson, Janette Young, Pauleen Bennett

**Affiliations:** 1 School of Psychology and Public Health, La Trobe University, Bendigo, VIC 3552, Australia; 2 OHAIRE, Comparative Pathobiology, Center for the Human Animal Bond, College of Veterinary Medicine, Purdue University, West Lafayette, IN 47907, USA; 3 Human-Animal Interaction, Department of Animal and Agriculture, Hartpury University, Gloucester GL19 3BE, UK; 4 School of Food and Agriculture, University of Maine, Orono, ME 04469, USA; 5 Dogs Trust, London EC1V 7RQ, UK; 6 Dogalov Human Support Initiative, Abuja 900108, Nigeria; 7 Centro de Estudios en Bienestar y Convivencia Social, Facultad de Psicología, Universidad del Desarrollo, Santiago 7610658, Chile; 8 Institute for Human-Animal Connection, University of Denver, Denver, CO 80208, USA; 9 EASE Working Group, College of Social Sciences and International Studies, University of Exeter, Exeter EX4 4PY, UK; 10 College of Education, University of Washington, Seattle, WA 98195, USA; 11 Department of Veterinary Medicine, Nippon Veterinary and Life Science University, Musashino, Tokyo 180-8602, Japan; 12 Department of Social Work, Beaver College of Health Sciences, Appalachian State University, Boone, NC 28607, USA; 13 Sports and Exercise Medicine, Biomedicine, University of Eastern Finland, Yliopistonranta 1, 70600 Kuopio, Finland; 14 Department of Anthrozoology, University of Exeter (alumnus), Exeter EX4 4PY, UK; 15 Child and Youth Studies, Brock University, St. Catharines, ON L2S 3A1, Canada; 16 Cork Pet Behaviour Centre, P85 YF58 Cork, Ireland; 17 Dogwood Therapy Services, Albuquerque, NM 87120, USA; 18 Animal Assisted Interventions International, 6537 HN Nijmegen, The Netherlands; 19 Department of Animal Sciences, Teikyo University of Science, Uenohara, Yamanashi 409-0193, Japan; 20 Illawarra Health and Medical Research Institute, University of Wollongong, Wollongong, NSW 2522, Australia; 21 School of Health, Medical & Applied Sciences, Central Queensland University, Melbourne, VIC 3000, Australia; 22 Professional Association of Therapeutic Horsemanship International, Denver, CO 80233, USA; 23 Risk Frontiers, St Leonards, NSW 2065, Australia; 24 Faculty of Law, Macquarie University, Sydney, NSW 2019, Australia; 25 Boynton Health, Office of Student Affairs, University of Minnesota, Minneapolis, MN 55455, USA; 26 University Centre Sparsholt, University of Winchester, Sparsholt, Winchester SO21 2NF, UK; 27 Dogs for Life, Caulfield South, VIC 3162, Australia; 28 Halcyon CIC, Worcestershire WR5 3LF, UK; 29 Virginia-Maryland College of Veterinary Medicine, Virginia Polytechnic Institute and State University, Blacksburg, VA 24061, USA; 30 The Program for Animal-Assisted-Psychotherapy, Tel-Hai College, Upper Galilee 1220800, Israel; 31 Department of Human Development, Washington State University, Pullman, WA 99164, USA; 32 Animal Therapies Ltd., Gold Coast, QLD 4209, Australia; 33 Management and Organizational Studies, Huron University College at Western University, London, ON N6G 1H3, Canada; 34 Equine Therapy Unit, ChildVision National Education Centre for Blind Children, D09 WKOH Dublin, Ireland; 35 Independent Researcher, Bedford MK40 2DU, UK; 36 The Canadian Institute of Animal-Assisted Interventions, Montréal, QC H3V 1C7, Canada; 37 Faculty of Psychology and Educational Sciences, Open University the Netherlands, 6419 AT Heerlen, The Netherlands; 38 Department of Psychology, Bemidji State University, Bemidji, MN 56601, USA; 39 School of Law & Social Justice, University of Liverpool, Liverpool L69 3BX, UK; 40 Center for Human-Animal Interaction, School of Medicine, Virginia Commonwealth University, Richmond, VA 23298, USA; 41 Independent Researcher, Toronto, ON M4E 3L9, Canada; 42 Stroke Research Programme, The Queen Elizabeth Hospital & Basil Hetzel Institute for Translational Health Research, Woodville South, SA 5011, Australia; 43 Center for Human Animal Psychology, Department of Communication and Psychology, Aalborg University, 9000 Aalborg, Denmark; 44 School of Veterinary Medicine, University of California, Davis, CA 95616, USA; 45 Clinical Animal Behaviour, Well Connected Canine, York YO24 3HG, UK; 46 Faculty of Social Science, University of Stirling, Stirling FK9 4LA, UK; 47 School of Nursing, Midwifery & Health Systems, University College Dublin, D04 V1W8 Dublin, Ireland; 48 Department of Anthropology, University of Nevada, Las Vegas, Las Vegas 89183, NV, USA; 49 Orygen Centre for Excellence in Youth Mental Health, University of Melbourne, Melbourne, VIC 3000, Australia; 50 Lead The Way Institute Ferntree Gully, Boronia, VIC 3156, Australia; 51 Hellenic Institute of Canine & Feline Behaviour, 15122 Athens, Greece; 52 MiMer Centre, 247 95 Torna Hallestad, Sweden; 53 Clinical Animal Behavior Service, Veterinary Medical Teaching Hospital, Chungbuk National University, Cheongju 28644, Korea; 54 Occupational Therapy, Health Care Sciences, Wayne State University, Detroit, MI 48202, USA; 55 Department of Public Health, Baylor University, Waco, TX 76798, USA; 56 Department of Education and Childhood Studies, Swansea University, Swansea SA2 8PP, UK; 57 Department of Clinical Sciences, Tufts Institute for Human-Animal Interaction, Cummings School of Veterinary Medicine, Tufts University, North Grafton, MA 01536, USA; 58 Department of Veterinary Sciences, University of Pisa, 56124 Pisa, Italy; 59 Scottish SPCA (Scottish Society for the Prevention of Cruelty to Animals), Dunfermline KY11 8RY, UK; 60 Hong Kong Animal Assisted Therapy Association (HKAATA), Hong Kong, China; 61 Guide Dogs NSW/ACT, St Leonards, NSW 2065, Australia; 62 Independent Researcher, Cumbria CA17 4QD, UK; 63 Dyrebar Omsorg AS, 1458 Fjellstrand, Norway; 64 Society for Companion Animal Studies, Godmachester, Cambridgeshire PE29 2BQ, UK; 65 Institute for Culture and Society, School of Social Sciences, Western Sydney University, Penrith, NSW 2751, Australia; 66 Department of Population Medicine, Ontario Veterinary College, University of Guelph, Guelph, ON N1G 2W1, Canada; 67 Human-Animal Bond in Colorado, School of Social Work, Colorado State University, Fort Collins, CO 80526, USA; 68 Northumbria Law School, Northumbria University, Newcastle upon Tyne NE1 8ST, UK; 69 Solihull College and University Centre, Solihull B91 1SB, UK; 70 The Royal (Dick) School of Veterinary Studies, The University of Edinburgh, Midlothian EH25 9RG, UK; 71 School of Health, Medical & Applied Sciences, College of Psychology, Central Queensland University, Rockhampton, QLD 4702, Australia; 72 Moray House School of Education and Sports Science, University of Edinburgh, Edinburgh EH8 8AQ, UK; 73 Centre for Urban Transitions, Swinburne University of Technology, Melbourne, VIC 3000, Australia; 74 Faculty of Health, Education and Society, The University of Northampton, Northampton NN1 5PH, UK; 75 Independent Researcher, Sunbury, VIC 3429, Australia; 76 School of Education, Communication and Language Sciences, Newcastle University, Newcastle upon Tyne NE1 7RU, UK; 77 Allied Health and Human Performance, University of South Australia, Adelaide, SA 5000, Australia

**Keywords:** companion animal, assistance animal, service animal, facility animal, therapy animal, emotional support animal, educational support animal, visiting animal, human–animal interaction

## Abstract

**Simple Summary:**

Although animals are being employed for a growing number of roles to support people, the terms used to describe those animals (e.g., “therapy animal” and “emotional support animal”) can be confusing. The same term may be used to describe different types of work, or the same role can be described with different terms. This paper presents the results of a collaboration between over 100 researchers, practitioners, and end users of animal-based supports from all over the world. We created working definitions for the following nine terms: “assistance animal”, “companion animal”, “educational/school support animal”, “emotional support animal”, “facility animal”, “service animal”, “skilled companion animal”, “therapy animal”, and “visiting/visitation animal”. In this paper, we describe the defining characteristics of each animal type and how it is different from the other types. We recommend phasing out the terms “skilled companion animal” and “service animal”, because they are similar to other terms. We discuss how our definitions may be received in different parts of the world.

**Abstract:**

The nomenclature used to describe animals working in roles supporting people can be confusing. The same term may be used to describe different roles, or two terms may mean the same thing. This confusion is evident among researchers, practitioners, and end users. Because certain animal roles are provided with legal protections and/or government-funding support in some jurisdictions, it is necessary to clearly define the existing terms to avoid confusion. The aim of this paper is to provide operationalized definitions for nine terms, which would be useful in many world regions: “assistance animal”, “companion animal”, “educational/school support animal”, “emotional support animal”, “facility animal”, “service animal”, “skilled companion animal”, “therapy animal”, and “visiting/visitation animal”. At the International Society for Anthrozoology (ISAZ) conferences in 2018 and 2020, over 100 delegates participated in workshops to define these terms, many of whom co-authored this paper. Through an iterative process, we have defined the nine terms and explained how they differ from each other. We recommend phasing out two terms (i.e., “skilled companion animal” and “service animal”) due to overlap with other terms that could potentially exacerbate confusion. The implications for several regions of the world are discussed.

## 1. Introduction

In recent years, animals have been increasingly recruited to support [1,2] people who have specific support needs or are in vulnerable or marginalized positions (e.g., children and adolescents, elderly, and people with disabilities, as defined by the United Nations [3]). These roles include, but are not limited to, animals who live with and assist a person with a disability, animals working in therapeutic or learning settings to benefit clients/patients/students, animals visiting residential facilities to improve well-being among residents, animals visiting schools to improve learning outcomes for children, and animals living with and supporting a person who has a diagnosed mental health condition. Herein, we refer to these animals as animals with a “support role” or animals “who support people”. Despite, or perhaps because of, the rapidly growing interest in animals working in these roles, service providers, researchers, and beneficiaries of these animal-based supports sometimes use different terms to describe the same role, or the same term to describe different roles.

This lack of clarity can cause confusion regarding the animal’s actual working role, whether any legal protections are afforded to the owner/handler of the animal [4], and the training standards implicit in the role. For example, an “assistance animal” in Australia [5] or the United Kingdom (UK) [6] is functionally the same as a “service animal” in the United States (US) [7]; that is, an animal living with and highly trained to mitigate the impacts of the owner’s disability, and with legal protections that are not afforded to most animals, namely to enter public spaces when with their handler. There also appears to be confusion about the difference between a psychiatric assistance animal for a person with a mental-health-related disability and an emotional support animal [8], who typically requires no special training [9]. This is complicated by the fact that some of the legal protections afforded to assistance animals in many parts of the world have been extended to emotional support animals in some jurisdictions, despite the lack of training requirements for the animals [8]. The term “therapy animal” is used by some training organizations to mean an animal trained to participate in activities such as visiting an aged care facility to bring enjoyment to the residents [10], by others to exclusively mean an animal that is part of a structured, goal-directed therapy program [11], and by others to refer to animals working in psychiatric assistance roles [12]. Such confusion makes it difficult to understand the type of support role that a particular animal fulfills.

To address these terminology issues, there have been several attempts to provide clarity around the types of activities in which animals may be involved. For instance, in 2018, the International Association for Human–Animal Interaction Organizations (IAHAIO) published recommended definitions for terms such as animal-assisted interventions, animal-assisted activities, animal-assisted therapy, animal-assisted learning, and animal-assisted coaching [13], with Animal Assisted Intervention International (AAII) publishing similar definitions for these roles [14]. Similarly, Wood et al. [15] provided suggested definitions for equine-assisted services in the US. While these papers gave useful guidance on how to define and name activities incorporating animals, neither defined the terms used to describe the animals themselves (e.g., “therapy animal” and “assistance animal”). In 2013, Parenti et al. [9] attempted to clarify existing terms by providing clear and operationalized definitions for animals in working roles. However, Parenti et al. [9] considered all working animals to be “assistance animals”, including military and police dogs, and dogs in racing and other sports. This suggested terminology is different from how assistance animals are conceptualized and legally defined in the US and in other parts of the world. Furthermore, the anthrozoology literature is generally biased toward wealthy countries, especially those in Europe and North America, along with Australia, New Zealand, and Japan; however, this is starting to change over time [16]. Less is known about animals working in support roles in other parts of the world (e.g., South America, Africa, and parts of Asia) and the terminology typically used to describe them.

Given the speed with which animal-based support roles are evolving, an update on terminology for animals working in support roles, with an international focus, is necessary to provide guidance for industry, researchers, and beneficiaries of these types of support around the world. The aim of this commentary is to provide clear operationalized definitions for animals who support people. The definitions provided in this paper were refined from existing draft definitions developed from an international workshop of leading experts in the field. The draft definitions and the associated review of the literature that informed them are available in Howell et al. [17].

### A Note on Standards

This paper defines the roles of animals who provide support to people. For standards of practice associated with each role, it is extremely important that organizations and individuals seek out and adhere to the professional standards for the country and individual discipline in which they practice. Unfortunately, there is evidence of great variation in practice in animal-assisted programs in the US [18], and this is likely to be the case elsewhere, too. It is beyond the scope of this paper to establish standards for each of the terms defined. Instead, we recommend relying on publicly available standards published by professional bodies. For instance, Assistance Dogs International provides training standards for assistance dogs [19]. Within Europe, the European Standards Agency, TC/452, is currently working to establish a European standard for assistance dogs. For visiting/visitation animals, Pet Partners provides information about its standards on their website [20]. There are also therapy animal organizations that publish ethical guidelines on their websites, such as the Federation of Horses in Education and Therapy International [21]. IAHAIO has published international guidelines for the care, training, and welfare requirements of animals in animal-assisted interventions [13], and AAII has published standards and competencies for animals in those roles [22]. For the purposes of the definitions provided in this paper, we consider the training standards inherent for each animal type and species, including no training at all, high standards of training (e.g., temperament tests and obedience training), or advanced training (e.g., trained for disability support tasks and public access).

## 2. Materials and Methods

### 2.1. Participants

A total of 137 experts in anthrozoology attended a workshop at the 2018 and/or 2020 International Society for Anthrozoology (ISAZ) conference, the leading international organization for research on human–animal relationships. The workshops aimed to create clear operationalized definitions of commonly used terms to describe animals who support people. The 2018 ISAZ conference was held in Sydney, Australia, and the workshop was attended by 43 participants. The 2020 ISAZ conference workshop was held virtually, with 101 participants. Seven experts attended both conference workshops. Participants from five continents attended the workshops.

### 2.2. Materials and Procedure

Ahead of the 2018 and 2020 ISAZ conferences, abstracts were submitted by authors T. Howell and P. Bennett and accepted by the conference organizers to facilitate workshop-style symposia to define common terms for animals in support roles. ISAZ conference delegates were able to attend the workshops at no extra cost beyond the typical ISAZ registration fees.

The participants in the 2018 ISAZ workshop were provided with a worksheet in which they were asked to consider how the following terms should be defined: “assistance animal”, “service animal”, “emotional support animal”, “facility animal”, “therapy animal”, “visitation animal, companion animal”, and “skilled companion animal”. These terms were selected from a preliminary review of peer-reviewed literature, Australian federal- and state-based legal statutes, and websites (e.g., “assistance animal” provider organizations and training organizations for “therapy animals”; for further details, see Reference [17]. To aid participants, an information sheet was provided which gave an overview of common uses of each term (see Supplementary Materials). Participants discussed the terms in 6-person groups for about 60 min before discussing with the wider group for about 20 min. The workshop was not audio-recorded, but the facilitators took notes to capture the key points as the workshop progressed, and participants supplied handwritten notes on the worksheets. These were used to create draft definitions [17].

At the 2020 workshop, participants were provided with the 2018 draft definitions and review of the existing literature [17]. They discussed the draft definitions during two one-hour virtual meetings held by using the Zoom online videoconferencing platform (Zoom Video Communications, San Jose, CA, USA). Participants also used the Jamboard online whiteboard (Google, Mountain View, CA, USA) and MS Teams online collaboration platform (Microsoft Corporation, Redmond, WA, USA) to provide feedback outside of the Zoom meetings.

All participants in the two workshops were invited to be co-authors on the publication arising from the discussion. Representatives from peak bodies (e.g., Society for Companion Animal Studies and Professional Association of Therapeutic Horsemanship International) were also invited to provide feedback on the draft definitions and join the publication as co-authors. All co-authors were expected to contribute to the publication by providing feedback on the definitions and/or entire manuscript, assisting with analysis of feedback and/or writing a section of the paper.

### 2.3. Analysis

The lead author combined the written feedback from MS Teams and Google Jamboard with transcripts and chat box posts from the recorded Zoom meetings from the 2020 workshop. Using MS Excel (Microsoft Corporation, Redmond, WA, USA), she analyzed the text to determine the main themes in the feedback from the draft definitions. Two co-authors, L. Nieforth and C. Thomas-Pino, independently coded 25% of the feedback to confirm consistency in interpretation.

## 3. Results—Recommended Definitions

The following nine recommended definitions, listed in alphabetical order, are based on our consideration of definitions provided in legal statutes, peer-reviewed scientific publications, industry publications, and by the ISAZ workshop participants. As the field of human–animal interaction continues to advance both in practice and in research, these definitions may be subject to change. Most, but not all, of these terms refer to animals employed in a particular working role (i.e., working animals). The terms which do not refer to a specific type of work are included because they have previously been used in some contexts to describe an animal in a working role, or there is sufficient confusion about the type of support they provide that it is necessary to clarify the definition.

When considering animals who provide support for a person with a disability, as opposed to a vulnerable person who does not have a disability, we adopted the World Health Organization (WHO)’s definition of disability. According to the WHO, “disability results from the interaction between individuals with a health condition with personal and environmental factors including negative attitudes, inaccessible transportation and public buildings, and limited social support” [23].

In roles where there are no theoretical restrictions on the species employed, only species and individuals whose well-being will not likely be compromised by performing these roles should be considered. Similarly, only species which can be legally owned in the relevant jurisdiction should be employed. In all cases, the animal’s well-being must be a primary consideration both during and outside of working situations. We recommend that the animal types listed below be considered volunteers participating in benefitting human health and/or well-being, rather than being considered assistive tools in a subservient role.

In the definitions provided below, the term “handler” means a person who is responsible for overseeing the work performed by a working animal. The handler may also be the direct beneficiary of the animal’s work. The term “owner” refers to the person who has legal ownership of the animal, regardless of working status. In general, we use the term “handler” in the context of working animals, and “owners” in the context of animals without a formal working role.

### 3.1. Assistance Animal

We recommend using this term to describe ***an animal who performs at least one identifiable task or behavior (not including any form of protection, comfort, or personal defense) to help a person with a disability to mitigate the impacts of that disability, and who is trained to a high standard of behavior and hygiene appropriate to access public spaces that are prohibited to most animals.*** This should be considered an umbrella term that encapsulates (but is not necessarily limited to): guide animals; hearing animals; mobility assistance animals; psychiatric assistance animals; assistance animals for developmental/intellectual disabilities (e.g., autism assistance); and medical alert animals, such as seizure and diabetes alert animals.

An assistance animal typically lives with the person being supported. The types of tasks that an assistance animal is trained to perform will vary depending on the person’s disability and individual needs. For this reason, it would be impossible to provide a comprehensive list of all the potential tasks an animal may need to perform. We recommend that there be a requirement for the animal to perform at least one identifiable task or behavior, which may or may not be dependent on a voluntary verbal cue from the handler. Examples of assistance-animal tasks or behaviors are as follows:

- A guide animal for a person with a vision impairment helps its handler navigate around the neighborhood, avoiding stepping onto the street and into oncoming traffic, and avoiding potholes and other hazards on the walking path.
- A person with a psychological disability who becomes disoriented and needs to be taken home. In this case, the person may not be in a position to request help from the animal—the animal must be trained to be able to recognize the need and perform the task.
- An assistance animal for a person on the autism spectrum who is trained to lie on the person when that person is extremely distressed, providing a tactile stimulus that helps ground the person to help them calm down.
- A medical alert animal who alerts its handler with diabetes, or the handler’s carer, of a potentially dangerous fluctuation in their blood glucose levels. The animal may also be trained to bring necessary supplies (e.g., test kit) to assist the person.

Some assistance animal tasks are context-dependent, rather than dependent on voluntary cues from the handler, but they mitigate the impacts of the handler’s disability (e.g., waking a person with post-traumatic stress disorder from a nightmare). Therefore, these should be considered appropriate tasks in this context. Our examples are not intended to be comprehensive, and there are other examples of context-specific, in addition to verbal-cue-specific, tasks that an assistance animal could be taught to perform.

### 3.2. Companion Animal

This term should generally be considered to refer to ***a pet animal with no specialized employment training***. The owners may enjoy benefits to their well-being or mental health due to the human–animal relationship, but well-being improvements or other positive outcomes are not required for the animal to be considered a companion animal.

### 3.3. Educational/School Support Animal

We recommend using this term to describe ***an animal working in educational settings/schools that engages in goal-directed, structured programs, or services, with outcomes that are educational or developmental in nature***. The work of such animals should be overseen by a qualified educator or pedagogue. Educational outcomes may pertain to animal care and welfare, the development of life skills, or curricular outcomes such as literacy and reading. Training standards for school support animals should be high since they work in schools or other educational settings. All interactions with school support animals should be facilitated by an educator/handler who is knowledgeable about that species.

Some animals live or work in schools but are not integrated into goal-directed, structured programs, so these are not educational support animals. The term used to describe these animals should be based on the work that they perform. For example, an animal engaged in a therapy or treatment aimed at improving mental or physical health in school settings, but overseen by a healthcare professional, would be a therapy animal. Alternatively, a well-trained animal–owner team visiting on a non-professional/volunteer basis would be a visitation animal, while an animal living in a school purely for student companionship would be a companion animal.

### 3.4. Emotional Support Animal

This legal term was created in the US and provides any person with a disability the right to have ***an animal who lives with and provides emotional benefit and/or support for the person, as confirmed by an appropriate qualified health care professional.*** Disability is defined extremely broadly in the Americans with Disabilities Act (2008 Amendment), which does not mention animals, but provides for “reasonable accommodation” for anyone with a disability, thus serving as the basis for assistance animals (termed “service dogs” in the US) and emotional support animals. In contrast to assistance animals, US emotional support animals need not perform tasks and do not require specific training for disability support. Therefore, owners with emotional support animals do not have legal public access rights in the US, apart from the US Fair Housing Act, which governs housing regulations. Most countries do not have this category of working animal.

Emotional support animals are often indistinct from companion animals, except that the owner must have a disability and experience some alleviation of symptoms because of his/her animal. Some protections are afforded to emotional support animals in the US, such as approval for the animal to reside in rental housing when the landlord does not permit pets; however, there is an exception for those that cause a health or safety hazard for anyone else on the property.

Emotional support dogs are not legally recognized in England, in contrast to assistance dogs, which enjoy protection under the Equality Act 2010. Therefore, where there is a “no pet” covenant in a residential lease, a landlord is under a duty to make a reasonable adjustment for an assistance dog (i.e., the dog is allowed to live with the tenant with a disability), but not an emotional support dog. This can lead to a person with an emotional support dog having to relinquish the dog that provides emotional support, or be evicted from his/her home [24].

Due to the absence of legal protections for emotional support animals in some countries or confusion around the legal protections in others, we recommend removing blanket pet bans from rental policies and that landlords be required to give serious consideration to allowing pets where possible. This is consistent with a growing policy trend. For example, in the Australian state of Victoria, a 2020 reform to state legislation means that landlords can only refuse pets with the consent of the Victorian Civil and Administrative Tribunal [25]. In Queensland, Australia, from October 2022, landlords will only be able to refuse pets on prescribed reasonable grounds, including health and safety, as defined in the legislation [26]. Similar laws are being canvased in England, where the recent Policy Paper “A fairer private rented sector” proposes that tenants have “the right to request a pet in their property, which the landlord must consider and cannot unreasonably refuse” [27]. Removing blanket pet bans in housing policy would negate the need in the USA to differentiate emotional support animals from companion animals when they can serve the same function for a person with a disability. Internationally, the intersection of housing rights and the right to live with companion or support animals varies widely across housing tenures, sectors, and living arrangements. In part, this is due to definitional issues around what constitutes an assistance animal, and in part due to restrictive housing policies and practice around pet ownership broadly [25,28,29]. Because there is no special training required for emotional support animals, we also recommend that public access rights not be applicable for emotional support animals, and instead be reserved exclusively for assistance animals who have been independently deemed temperamentally suited and trained for appropriate behavior in public and can perform identifiable tasks or behaviors that mitigate the impact of the disability for the owner.

### 3.5. Facility Animal

This term is sometimes used to denote ***an animal trained to work in a particular facility (e.g., a hospital or school) or context (e.g., legal settings)***. The animal may or may not live on-site at the facility. Because this term is used so widely to describe educational/school support, therapy, and visitation animals, we recommend phasing it out in most contexts and using more specific terms (e.g., “therapy” and “visitation”) instead. An exception is animals working in legal settings to provide comfort for vulnerable witnesses, because the nature of their work does not fit under any other existing categories. These animals are present, often throughout the entire legal process, to support people providing evidence to prosecutors and/or in court. We recommend that the term “justice facility animals” be used to describe animals working in these settings. This term, in particular, may need to be revisited in the future as the contexts and roles played by animals increase.

### 3.6. Service Animal

This term should generally be considered ***synonymous with “assistance animal”*** when used in some North American (e.g., US) and European (e.g., Denmark) countries, especially when referring to dogs working in disability assistance roles. Because having two terms to refer to the same concept is confusing, we recommend phasing out the use of “service animal”. However, we recognize that legislation in some North American countries typically refers to “service animal” in the context of an animal providing disability support, so phasing it out of these countries may be challenging. In Europe, the European Committee for Standardization is currently working toward standards for assistance dogs, including standardizing the term “assistance dogs” [30]. For this reason, in the future, the term “assistance animal” may replace “service animal” in European contexts.

In the UK, the term “service animal” refers to an animal working in the military or with police [31]. To avoid confusion, we recommend phasing this term out in the UK and replacing it with another term, such as “military animal” or “police animal”.

### 3.7. Skilled Companion Animal

This is a term used by some assistance animal provider organizations to describe ***animals who are trained to help an individual person with a disability, and who are trained to a high standard of behavior and hygiene appropriate to access public spaces that are generally off-limits to animals, but who are handled by a facilitator rather than the individual with the disability***. The facilitator is often a parent, caregiver, or spouse of the adult or child with a disability and serves as the primary handler of the skilled companion animal. For example, an autism assistance dog may be placed with a child with autism, but primarily handled by the child’s parent at home and in public settings. While differentiating these animals from assistance animals is sensible given their somewhat different public access rights and handling, the use of this term is confusing because it is very similar to “companion animal”, which refers to animals that do not have special training or public access rights. Similar to all assistance animals, these types of animals only have public access rights when working and when accompanied by a handler. Therefore, we recommend that animals who fit these criteria be considered assistance animals and that the “skilled companion animal” category be phased out.

### 3.8. Therapy Animal

We recommend adapting the definition of Animal-Assisted Therapy provided by IAHAIO [13] and AAII [14] to define a therapy animal as ***an animal who is included into the work of a qualified health professional in the provision of a structured, goal-directed treatment***. In these programs, the animal may be employed by the professional to help the client meet specific goals (e.g., gait improvements in children with mobility impairments during therapy sessions with horses) [22]. The animal should not be considered the therapist; instead, interactions with the animal are incorporated into the work of a qualified professional to offer therapeutic benefit for the client.

Our recommended definition may exclude the use of this term for animals working in other contexts, such as animal-assisted social work, if it does not constitute therapy per se. Similarly, animals working with volunteers who are not engaging in goal-directed therapies are also sometimes referred to as therapy animals [32,33]—we do not recommend this term be applied to such situations. For instance, horses who are ridden in adaptive riding or therapeutic riding lessons with the purpose of providing access to horsemanship for people with disabilities should not be called therapy horses. The term “therapy horse” should be reserved for when interactions with horses or their movement is part of treatment. We recognize the challenges associated with narrowly defining this term but maintain its importance for improving clarity among the working roles fulfilled by these animals.

### 3.9. Visitation or Visiting Animal

We recommend using this term for ***companion animals that have suitable characteristics (e.g., are calm, have appropriate behavior and activity levels for the job, and are friendly) and are trained for public visitation by humans who volunteer to take them into facilities to bring enjoyment or other improvements in well-being to the people in those facilities (e.g., hospitals, elderly care facilities, and schools).*** The owners do not need to have any particular professional qualification (e.g., psychologist and occupational therapist) or registration with a relevant professional body, but the animal–human teams should ideally be evaluated and certified/registered by an organization that provides training for the teams, oversight for ongoing health checks, and adequate insurance to cover any potential issues when visiting facilities on a volunteer basis. Some visitation animals may also perform therapy work, and therapy animals may also perform visitation sessions. Therefore, the appropriate terminology should be applied to the specific context in which the animal is working at a particular time. The same animal could be a therapy animal one day, a companion animal the next day, and a visiting/visitation animal the following day.

A summary of these terms is provided in Table 1.

**Figure 1:**
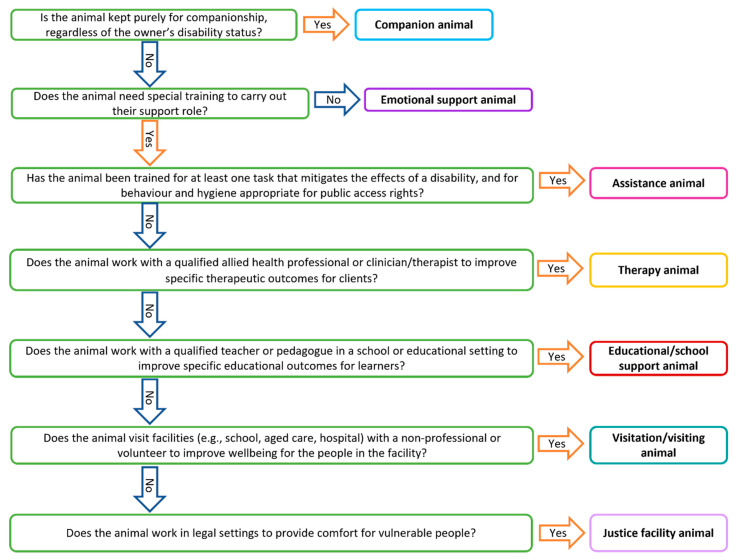
Flow diagram of relationships between various animal roles.

A flowchart showing the relationships between the roles is available in Figure 1.

## 4. Discussion

The aim of this paper is to provide updated definitions for commonly used terms describing animals working to support people. A large group of international collaborators worked together to create definitions that will be relevant in many areas of the world and in many different contexts.

In this paper, we recommend that nine terms currently in use be consolidated into seven terms. In particular, “service animal” and “skilled companion animal” should be phased out to address some of the confusion associated with the overlap among these and the term “assistance animal” in the field of human animal interactions. We concede that this might prove challenging in some North American and European countries where the use of “service animal” relates to key legislation. However, phasing out this term is an important step toward achieving greater international clarity and specificity in defining an “assistance animal”, and can offer guidance to researchers, service providers, legal experts, policymakers, and beneficiaries of animal supports around the world. Given the challenges associated with our recommendation, in the near term, “service” and “assistance” animals should be considered synonymous.

We also recommend phasing out the term “facility animal” in most contexts, as the specific roles undertaken by animals currently called “facility animal” usually reflect another existing category (e.g., an animal with no special training who joins its owner at work in an aged care facility and occasionally interacts with the residents would be a companion animal). The exception to this is animals working in legal settings to provide comfort for staff and vulnerable witnesses. The nature of this work is so different from any of the definitions we provided for other terms that we recommend the term “facility animal” be used exclusively for this purpose and that it ideally be renamed “justice facility animal” for the sake of clarity. Given the rapidly evolving nature of animal-based support work, it is possible that this term will need to be expanded to include animals working outside of legal contexts in the future.

By providing clarity for terms, we strengthen our commitment to highlighting working animals as welcome members in research, education, family, and community-based contexts, and as reciprocal partners in a process that supports well-being among people with support needs or in marginalized or vulnerable situations [34]. In this way, we recognize that working animals are not simply “assistive tools” in a subservient role to be used to provide a service, but rather that all animals are actively and continually engaged in shaping the quality of their interactions with people. Working animals can offer well-being support to people across varied settings and through both formal and informal interactions.

Not only does this approach correspond with recent calls for a repositioning of working animals that highlights reciprocity in animals’ interactions with people who have support needs or are in marginalized or vulnerable situations, but it also foregrounds an increasing focus on animal welfare concerns [35,36,37]. By foregrounding animal agency, we are better attuned to animals’ motivation, or lack thereof, to participate in working roles supporting people. When animal welfare is a concern, we might consider opportunities to interact virtually with animals to support well-being among people with support needs (e.g., observing an animal via videoconferencing software); this is one unobtrusive space where working animals can be engaged without intrusion and their welfare respected. It is important that researchers, service providers, and beneficiaries of animal supports alike consider issues of animal welfare and that we reposition the notion that animals are “used” for human well-being. Rather, all working animals are to be considered key stakeholders whose contribution to the positive outcomes arising from their interactions with people who have support needs can only be optimized when issues of animal welfare are considered and remain front and center [38,39,40,41,42]. For this reason, we do not recommend the use of any non-domesticated species in supporting people with support needs. Non-domesticated species are not likely to experience any benefits from ongoing interactions with humans; since it would be virtually impossible to ensure their welfare, they should not be considered for these working roles.

### 4.1. Elevate the Status of Companion Animals to Negate the Need for Emotional Support Animal Documentation

While companion animals can provide emotional support and contribute to the mental well-being of their owners, only those belonging to someone with a disability are eligible for emotional support animal status in the US. There is a considerable body of research to support the role companion animals play in reducing loneliness, providing emotional support, and alleviating some of the symptoms of mental illnesses [43,44,45,46]. However, the data are not consistent, suggesting that many factors are involved that may render the responsibility and stress of caring for a companion animal detrimental to mental and physical health [47,48]. Thus, it is not recommended to acquire an animal for the sole purpose of emotional support. However, people can and do develop strong bonds with companion animals that are akin to kinship bonds [49,50,51,52]. For such closely bonded individuals, being forced to relinquish their companion can have severe detrimental effects to their well-being [53,54]. Conversely, closely bonded individuals who refuse to relinquish their companion animals can experience detrimental consequences, such as housing insecurity [28]. However, only those with a disability may claim emotional support animal status for their companions and avoid choosing between a beloved animal and a roof over their head. The emotional support animal status enables individuals to remain living with their animals in rented accommodations, and often convinces businesses and institutions to allow them to be brought onto premises where companion animals are typically excluded, such as college buildings, airplane cabins, and crowded shopping malls. However, confusion over the meaning of emotional support animal [4] may lead to animals being brought into environments that they have not received training for and in which they are less able to cope. As the emotional support animal status requires no special training or screening, this could potentially be stressful or dangerous for the animal. A better solution would be a lifting of “no animals” rules on rental homes, as doing so would negate any need for the emotional support animal category, especially since changes to travel laws no longer permit emotional support animals on airlines in the US [55], where they were previously permitted.

### 4.2. Implications for Different Regions of the World

A limitation of previous attempts to define terms similar to the ones described in this paper (e.g., References [9,17]) is the lack of discussion about how these terms might be received in various parts of the world. Therefore, this section provides an overview of the implications for many major world regions, listed alphabetically. We attempted to be as inclusive as possible, but within each geographical region, there will certainly be variation in laws within individual countries and jurisdictions. Covering every country is beyond the scope of this paper. A table summarizing the key points is available below (see Table 2).

Our proposed definitions could impact different regions in various ways. In areas where the industry around animal-based supports is nascent, our definitions could provide a basis for further development as the industry grows. In these areas, it could prevent the adoption of unclear terms. In regions with established animal-related support industries, it could help clarify the differences between some common roles, especially “therapy animal”, “educational/school support animal”, and “visiting/visitation animal”. It is also hoped that the difference between a psychiatric assistance animal and an emotional support animal will be clarified.

### 4.3. Animal Welfare Considerations

It is beyond the scope of this paper to propose standards for the categories of animal defined; however, it is incumbent upon us to recommend a functional model for animal welfare and its implications for working animals.

Globally, the approach to animal welfare has been defined by the Five Freedoms: freedom from hunger and thirst; freedom from discomfort; freedom from pain, injury, and disease; freedom from fear and distress; and the freedom to express normal behavior [56]. This approach has been utilized in the creation of standards and legislation to safeguard welfare. However, there has been a global shift to harness the Five Domains (i.e., nutrition, environment, health, behavior, and mental state [35,36]) as a functional model to assess animal welfare.

The previously accepted concept of the Five Freedoms (i.e., that providing for an animal’s welfare required ensuring that negative states such as pain, fear, and distress were minimized and that the animal had the opportunity to perform normal behaviors) is now recognized as being outdated [36]. Animals are sentient and capable of feeling pain and experiencing negative states, and simply avoiding such states does not ensure that an animal has a life worth living or a good life [36]. Good welfare and having a life worth living also depend on the animal experiencing actively positive states. Positive states include opportunities for comfort, pleasure, interest, attachment, confidence, and a sense of being in control [36].

Regardless of personality, temperament, and training, all animals working in roles supporting people require support during challenging emotional exchanges, both before and after their formal work, and opportunities to take breaks and engage in meaningful leisure time to help make these more humane jobs [38,39]. We recommend that the Five Domains Model be the accepted welfare framework for application in all animal types defined in this paper.

## 5. Conclusions

Researchers, practitioners, and others with an interest in human–animal relationships provided working definitions for nine existing terms used to describe animals working in roles to support people with support needs or in marginalized and vulnerable situations. We recommend phasing out “skilled companion animal” and “service animal” due to overlap with our definition of “assistance animal”. We discussed implications for various regions of the world, making this the first paper, to our knowledge, to take an international approach to these definitions. We acknowledge that the roles that animals play in our lives will evolve over time, and these definitions may need to change along with them in the future. Similarly, the international approach, while novel, was preliminary. For instance, thorough consideration of the implications for individual countries and cultural groups within each country/region (e.g., First Nations peoples) was beyond the scope of this report. Further research is warranted to thoroughly investigate the implications of our proposed definitions in different parts of the world.

## Figures and Tables

**Table 1:** Summary of recommended definitions for commonly used terms describing animals working in support roles for people, in alphabetical order. Terms that we recommend phasing out are shaded, and a term that we recommend modifying is italicized.

Term	General Purpose	Training Standards *	Public Access **	Key Points
Assistance Animal	Lives with and supports a handler with a disability/disabilities (physical, developmental, intellectual, neurological, and/or psychological)	Advanced	Yes	Umbrella term for an animal typically living with a handler with a disability (or a family member who could serve as the handler) that has been trained to perform tasks that mitigate effects of that specific disability, with behavior and hygiene maintenance suitable for public access.
Companion Animal	Companionship	None	No	Synonymous with pet (i.e., an animal kept purely for companionship). Other benefits to well-being may be enjoyed by the owner, but this is not a requirement.
Educational/School Support Animal	Educational support—improve learning or developmental outcomes for students	High	No	An animal who works in educational settings with a handler to improve educational outcomes for participants. Educational activities must be structured, goal-directed, and overseen by a licensed teacher or pedagogue.
Emotional Support Animal	Emotional support, primarily in the home, for an owner with a diagnosed disability	None	No	Differs from assistance animal in training standards for public access and does not perform specific tasks to provide disability support tasks/behaviors.
*Facility* *Animal*	Depends on the specific role of the animal	High	No	Typically, an animal with training to work in a specific facility (e.g., a hospital) or type of facility (e.g., legal settings). Recommend mostly phasing this term out due to broad, vague nature of current use and overlap with other terms in most cases, with the exception of animals working in legal settings, which we recommend calling ***“justice facility animal”.***
Service Animal	Synonymous with assistance animal	Advanced	Yes	This term is commonly used to describe assistance animals in some North American and European countries. Recommend phasing out and using the term “assistance animal”.
Skilled Companion Animal	Disability support for an individual with a disability under the guidance of a facilitator	Advanced	Yes—when with facilitator	Term used by some assistance animal providers. Recommend phasing out and using the term “assistance animal”.
Therapy Animal	Improve specific therapeutic outcomes	High	No	Animal is integrated into therapy or treatment which must be structured, goal-directed, and overseen by a licensed healthcare professional trained in the relevant therapeutic field.
Visitation or Visiting Animal	Improve general quality of life, various settings (e.g., hospitals, aged care, residential care)	High	No	Well-trained animal–handler team, primarily performed on a non-professional or volunteer basis. Differs from therapy animal (above) as programs are unstructured with no specific therapeutic goals, although some participants may experience benefits to well-being.

* Training standards: none = no training of any kind required; high = training for appropriate temperament and behavior to interact with people who have specific needs; advanced = training for public access and disability support. ** Public access indicates whether the animal has the legal right to enter public places that are usually off-limits to animals (e.g., cafes, restaurants, banks, and national parks), depending on legal regulations in the jurisdiction.

**Table 2:** An overview of the implications of our proposed definitions for various regions of the world.

Region	Key Points
Africa	Inconsistent uptake of legal standardization throughout continent; IAHAIO, AAII, and SCAS beginning to operate in Africa, and globally accepted standards/definitions are provided; Our proposed definitions could be a useful guide for a developing industry.
Asia	Assistance dogs present in many countries, but with restrictions on which disabilities are supported (e.g., for Japan, guide, hearing, and mobility dogs; for South Korea, guide and hearing dogs; and Hong Kong and Singapore, guide dogs); In India, “service dogs” and “assistance dogs” both refer to our definition of “assistance animal”, but they are rare; In most parts of Asia, “therapy animal” may mean our definition of the term or our definition of “educational/school support” or “visiting/visitation” animal; In Japan, animal specialists working in animal-assisted programs, rather than human services or healthcare professionals; adoption of our definitions may promote participation of human specialists in animal-assisted programs; No known animal-based supports in Indonesia; one known guide dog in Malaysia.
Australia/New Zealand	In Australia, “assistance animals” are defined in legislation; the definition accords with our recommended definition; In New Zealand, “disability assist dog” is recognized in legislation; Emotional support animals not formally recognized; The term “therapy animal” used to describe our definition of “therapy animal”, but also our definition of “educational/school support” or “visiting/visitation” animal.
Europe	Variation between countries regarding terminology and standards; European Committee for Standardization working to establish standards for assistance dogs; their “assistance dog” agrees with our definition of “assistance animal”; The term “therapy animal” is used to describe our definition of “therapy animal”, but also our definition of “visiting/visitation” animal.
North America	In the United States (US), “service dog” is used consistently in legislation, although State of California uses “assistance animal”; accords with our definition of “assistance animal”; One use of term “assistance animal” in federal US legislation, but it refers to service dogs and emotional support animals; it does not agree with our definition of “assistance animal”; May be difficult to phase out “service animal” in US; In Canada, “service animals”, in provincial laws, are similar to “service dogs” in the US, but may be referred to as “service dog”, “guide dog”, “service animal”, and “assist animal”; In Canada, “police service dog” describes dogs working in the military, but it is not known whether this causes confusion around service dogs for disability; The term “therapy animal” used to describe our definition of “therapy animal”, but also our definition of “visiting/visitation” animal.
South America	Many countries refer to “assistance animal” in legislation, rather than “service animal”; Some countries have limits on disability types supported by assistance animals (e.g., in Peru and Brazil, only guide dogs are recognized in legislation); Some civic organizations are members of IAHAIO and adopt their terminology; The term “therapy animal” is used to describe our definition of “therapy animal”, but also our definition of “visiting/visitation” animal; No provision for emotional support animals.

## Data Availability

De-identified qualitative data can be made available by contacting the lead author.

## Supplementary Materials

The following supporting information can be downloaded at https://www.mdpi.com/article/10.3390/ani12151975/s1: Terminology—current usage.
