# Supplementary material for: Defining Terms Used for Animals Working in Support Roles for People with Support Needs
Source: Animals (Basel). 2022 Aug 4;12(15):1975. doi: 10.3390/ani12151975 (PMC9367407; doi:10.3390/ani12151975)
Supplement: Supplementary file 1 [file animals-12-01975-s001.zip › animals-1772907-supplementary.pdf]

Supplementary material: Terminology – Current usage

Assistance Animal

- Generally used as an umbrella term for various types of service animals (particularly when sources differentiate between guide, hearing, and other service animals).
- A few sources include **emotional support dogs and therapy animals** under this term.
    - o US Housing and Urban Development Fair Housing Act defines the term "assistance animal" as including those who provide "emotional support to individuals with disabilities who have a disability-related need for such support"
    - o Schoenfeld-Tacher 2017: "assistance animals can be viewed as an umbrella label, with sub-categories including, but not limited to, service animals, therapy animals, and emotional support animals"
- Australian Civil Aviation (1988) is the only one that uses the term "assistant" to refer only to dogs mitigating visual or hearing related disabilities.
- Australian Disability Discrimination Act 1999/2009, Section 9, defines an assistance animal as a dog or other animal that is:
    - o (a) accredited under a law of a State or Territory that provides for the accreditation of animals trained to assist a person with a disability to alleviate the effect of the disability; or
    - o (b) accredited by an animal training organisation prescribed by the regulations for the purposes of this paragraph; or
    - o (c) trained:

        (i) to assist a person with a disability to alleviate the effect of the disability; and

        (ii) to meet standards of hygiene and behaviour that are appropriate for an animal in a public place.

Service Animal

- Generally refers to **dogs** individually trained to perform work/tasks that mitigate their handler's disability including physical, sensory, psychiatric, intellectual, or other mental disability
    - o US Americans with Disabilities Act Title II and III specify that service animals must be dogs (and in special cases miniature horses)
- Sometimes sources distinguish "guide" and "signal" dogs from "service" dogs
    - o For example, ADI glossary: "dogs that work for individuals with disabilities other than blindness or deafness…"

  * Guide – provide mobility assistance to individuals with impaired vision

  * Hearing/Signal – provide assistance to individuals with impaired hearing

Emotional Support Animal

- Generally refers to an animal that mitigates an individual's psychological disability. No specific task/work training is required, but the individual must have a diagnosed disability. Can be any species.
- Sometimes used interchangeably with other terms:
    - o Parenti (2013) – "Common labels used for dogs include emotional support dogs, social therapy dogs, skilled companions, and home-help dogs."

    - Von Bergen (2015) – "ESAs (sometimes called therapy animals or support animals)"

Facility Animal

- Generally an umbrella term for dogs that go to healthcare facilities or in legal settings like courtrooms (whether with a volunteer for visitation or working with a professional in a therapy program)

Therapy Animal

- Appears to have two main, **conflicting** definitions:
    - Animal that plays a role in animal-assisted intervention within the context of a set treatment plan conducted by a healthcare professional, often in a clinical setting
        - definition consistent with those provided by IAHAIO, AVMA, Parenti 2013, Foreman 2017
    - Animal that visits facilities with a volunteer handler (usually owner) to "provide comfort and companionship by sharing the dog with patients in hospitals, nursing homes and other institutions and wherever else the Therapy Dog is needed" (Therapy Dogs International)
        - Definition consistent with those provided by Pet Partners (formerly Delta Society), Delta Society Australia, Therapy Dogs International
        - Seems to be no different from "visitation" animals
        - McCabe (2002) also uses a dog for social interaction, but their "therapy dog" lived full time at the aged care facility.

Visitation Animal

- Generally a term for animals that socialize with patients or students to provide comfort/companionship/social interaction. Often handler is pet owner.
- Overlapping definition with one of the uses of the term "therapy" animal.

Skilled Companion Animal

- Definition unclear.
    - Parenti (2013) – "Common labels used for dogs include emotional support dogs, social therapy dogs, skilled companions, and home-help dogs."
    - ADI Glossary – "A Skilled Companion Dog is a service dog trained to work or perform tasks with an adult or child with a disability under the guidance of an additional person, a facilitator"
    - Sometimes referred to as **'companion animal'** (e.g. Guide Dogs Victoria and Guide Dogs WA website)
